# Multi-view AI output variability as a teaching resource in a micro-course for thyroid TI-RADS interpretation among ultrasound residents: a randomized controlled trial

**DOI:** 10.3389/fmed.2026.1868341

**Published:** 2026-08-26

**Authors:** Chao Fu, Kefei Cui, Caifeng Si, Jing Yu, Yan Zhang

**Affiliations:** Department of Ultrasound, The First Affiliated Hospital of Zhengzhou University, Zhengzhou, Henan, China

**Keywords:** artificial intelligence in medical education, multi-view AI output variability, randomized controlled trial, TI-RADS, ultrasound residents

## Abstract

**Introduction:**

Thyroid TI-RADS interpretation requires integration of sonographic features across image planes, yet routine teaching often emphasizes the final category more than the cross-plane reasoning process. We evaluated whether a structured micro-course using multi-view AI output variability as a teaching resource could improve TI-RADS interpretation among ultrasound residents.

**Methods:**

In this single-center randomized controlled trial, 52 ultrasound residents were randomized 1:1 to either a structured micro-course using multi-view AI output variability as a teaching resource or time-matched conventional TI-RADS teaching. The primary outcome was participant-level TI-RADS interpretation accuracy at baseline (T0), immediately after teaching (T1), and 4 weeks later (T2). Secondary outcomes were inter-learner consistency, representative plane hit score, and self-efficacy. Justification quality in discordant cases was analyzed exploratorily.

**Results:**

Baseline characteristics were comparable between groups. Accuracy showed a significant group-by-time interaction [*F* (2, 100.0) = 10.86, *P* < 0.001]. There was no between-group difference at T0 (*β* = 0.08, 95% CI: −0.74 to 0.89; *P* = 0.851), but the intervention group performed better at T1 (*β* = 1.69, 95% CI: 0.88–2.51; *P* < 0.001). The between-group difference at T2 was not significant (*β* = 0.73, 95% CI: −0.08 to 1.55; *P* = 0.078). At T1, the intervention group also had a higher representative plane hit score (15.00 [12.00–17.00] vs. 13.00 [11.00–15.00]; *P* < 0.001), higher inter-learner consistency at T1 and T2 (ΔAC2 = 0.08 and 0.09, respectively; both 95% CIs excluded zero), and a greater increase in self-efficacy (*β* = 0.27, 95% CI: 0.09–0.45; *P* = 0.006). Exploratory analysis showed more favorable justification quality in the intervention group (OR: 10.26, 95% CI: 5.36–19.67; *P* < 0.001).

**Conclusions:**

A structured micro-course using multi-view AI output variability as a teaching resource improved immediate TI-RADS interpretation accuracy, but the between-group advantage was not sustained at 4 weeks, despite persistently higher inter-learner consistency. These findings support the short-term educational value of the structured micro-course rather than an independent effect of AI output variability and warrant evaluation of repeated or booster instruction.

## 1 Introduction

Thyroid Imaging Reporting and Data System (TI-RADS) provides a standardized framework for stratifying malignancy risk and guiding biopsy and follow-up decisions through assessment of composition, echogenicity, shape, margins, and echogenic foci (1–3). In practice, however, these features are not always equally visible across image planes (4). Accurate interpretation therefore requires learners not only to apply scoring rules, but also to compare views, judge which image best represents the lesion, and integrate partially discordant visual information into a final category (4, 5). This process can be difficult to teach because key aspects of image interpretation are often perceptual and tacit rather than fully formalized in explicit verbal rules (6).

In routine teaching, instructors often emphasize the final TI-RADS category, whereas explicit guidance on how to reconcile cross-plane feature differences is limited, partly because ultrasound teaching faculty are scarce (7, 8). From the perspectives of clinical reasoning and cognitive apprenticeship, effective instruction should make expert comparison, prioritization, and evidence integration visible to learners (9, 10). This need is especially relevant to thyroid ultrasound, because lesion composition, shape, margins, and echogenic foci may not be equally apparent across image planes (4, 5).

Previous studies of artificial intelligence-based computer-aided diagnosis (AI-CAD) in thyroid ultrasound have mainly evaluated diagnostic performance, risk stratification, or assistance for less experienced readers (11–14, 16). Systems such as S-Detect and AI-SONIC Thyroid generate feature-level assessments and TI-RADS categories from submitted images. Because these outputs depend on the selected image, different views of the same lesion may yield discordant feature assessments or categories (15). However, most educational applications have treated AI as a source of diagnostic support or feedback on a final answer rather than as a resource for teaching how to compare views and resolve discordant evidence.

In the present study, within-case differences in AI-generated feature assessments or TI-RADS categories across submitted views were termed multi-view AI output variability. Rather than using AI output as a reference standard, we used such variability as a discrepancy signal during faculty-guided discussion. The instructional premise was that visible differences between plane-level outputs could externalize otherwise tacit reasoning by prompting learners to compare views, identify possible sources of discordance, select a representative plane, and integrate evidence across views. This teaching-oriented use of AI output variability has received less attention than AI-assisted diagnostic performance, leaving uncertainty about whether it improves learners’ independent TI-RADS interpretation.

We therefore conducted a single-center randomized controlled trial to evaluate whether a structured micro-course using multi-view AI output variability as a teaching resource during faculty-guided case discussions improved TI-RADS interpretation accuracy among ultrasound residents. Associations with inter-learner consistency and representative plane hit score were also examined, while justification quality was treated as an exploratory post-instruction analysis.

## 2 Methods

### 2.1 Study design

This single-center superiority randomized controlled trial with 1:1 allocation was conducted within the standardized ultrasound residency program at The First Affiliated Hospital of Zhengzhou University. The classification method used for training and assessment in this study was the American College of Radiology Thyroid Imaging Reporting and Data System (ACR TI-RADS) (1, 3). PGY1–3 residents were stratified into two predefined training strata (PGY1–2 and PGY3) and randomly assigned to the intervention or control group using a computer-generated block randomization sequence prepared by an independent researcher. Allocation was concealed from the enrolling investigator through sealed opaque envelopes until assignment. Participants were enrolled by designated study personnel, and group allocation was implemented by a separate investigator; neither had access to the full allocation sequence beforehand. Outcome assessors for justification quality were blinded to study group. The trial was designed and reported in accordance with the CONSORT statement. Ethical approval was obtained from the Ethics Committee of The First Affiliated Hospital of Zhengzhou University (approval no. 2026-KY-0138). Written informed consent was obtained from all participants before enrollment.

Sample size was estimated *a priori* for the primary outcome, participant-level TI-RADS interpretation accuracy, using a two-group repeated-measures design. The calculation targeted the between-group difference in change from baseline to immediately after teaching. Assuming a moderate standardized difference in longitudinal change (Cohen's *d* = 0.60) (17), a two-sided *α* of 0.05, 80% power, and an assumed within-participant correlation of approximately 0.70 among repeated measurements, 26 participants per group were required.

### 2.2 Participants

Residents enrolled in the ultrasound residency program (PGY1–3) were recruited consecutively between January 1 and March 1, 2026. Eligible participants were required to have completed basic ultrasound training sufficient to recognize standard transverse and longitudinal thyroid planes. Residents were excluded if they had completed advanced thyroid-focused fellowship training or were simultaneously participating in intensive thyroid- or AI-focused training outside the study.

### 2.3 Interventions

**Figure 1:**
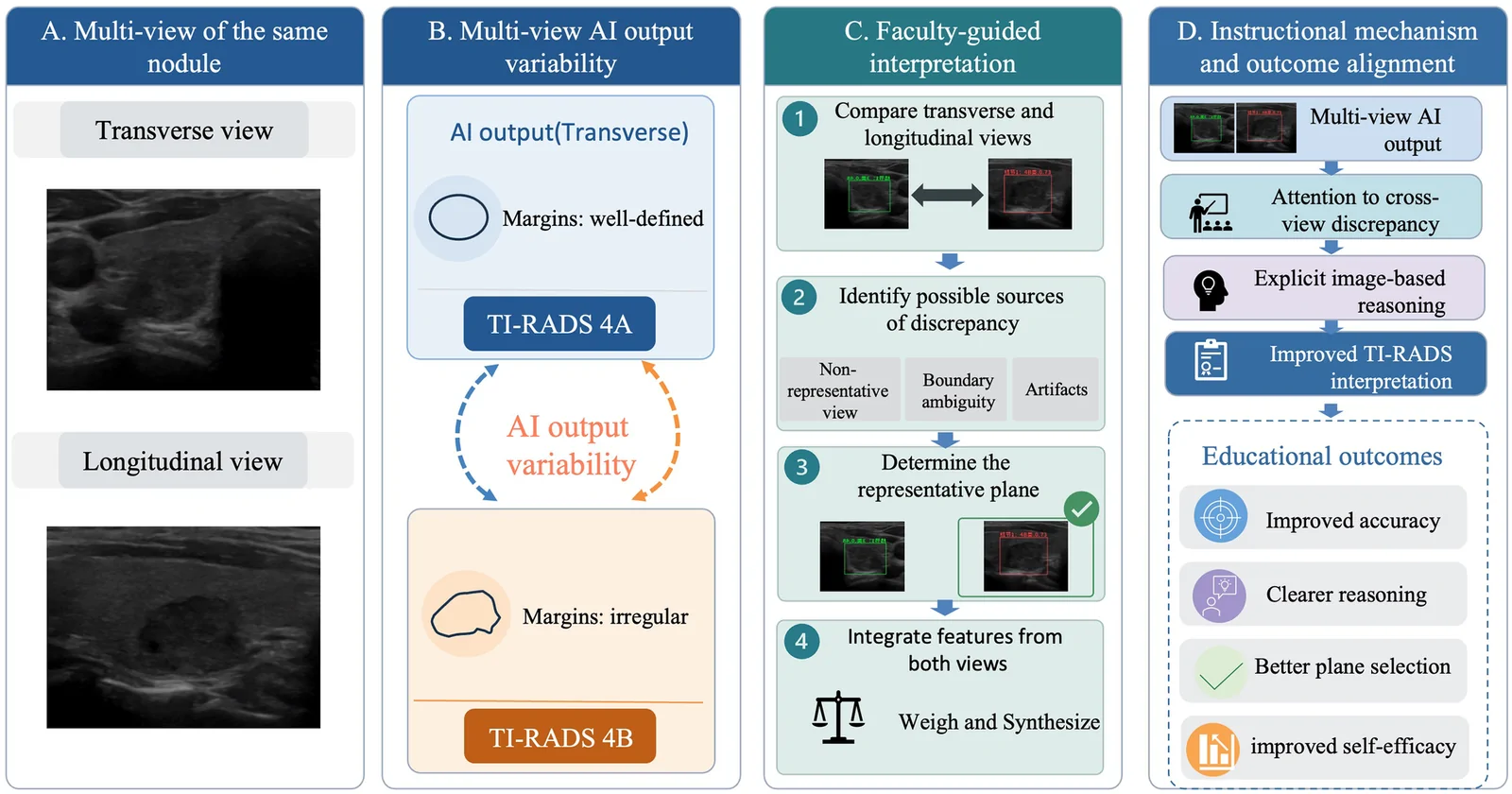
Proposed instructional pathway of using multi-view AI output variability in TI-RADS training. **(A)** Transverse and longitudinal ultrasound images show two orthogonal views of the same thyroid nodule. **(B)** Multi-view AI output variability. **(C)** Faculty guided residents through four sequential steps. **(D)** The proposed instructional pathway links exposure to multi-view AI output variability with attention to cross-view discrepancies, explicit image-based reasoning, and improved TI-RADS interpretation, with accuracy, reasoning clarity, representative-plane selection, and self-efficacy as educational outcomes. AI outputs served as discrepancy signals and did not constitute the reference standard or answer key. AI, artificial intelligence; TI-RADS, Thyroid Imaging Reporting and Data System.

Both groups attended a single 90-min instructor-led educational session delivered by the same senior faculty member, with identical session duration, learning objectives, and overall case-discussion format. Standardized slide materials and a prespecified facilitation checklist were used to maintain instructional consistency. The intervention contrast was defined by whether participants were exposed during teaching to plane-level AI outputs and structured prompts targeting cross-view discrepancy analysis. Figure 1 illustrates how multi-view AI output variability was used as a teaching resource in a micro-course for thyroid TI-RADS training.

#### 2.3.1 Intervention group

Participants in the intervention arm received a micro-course using multi-view AI output variability as the core teaching resource. During case discussion, residents reviewed multiple views of the same nodule along with the corresponding AI outputs for each view, including feature analysis and TI-RADS categorization.

#### 2.3.2 Control group

Participants in the control arm received a time-matched conventional instructor-led case-based TI-RADS teaching session. They reviewed static thyroid nodule cases and discussed TI-RADS feature identification and final categorization under faculty guidance, but were not shown plane-level AI outputs or given structured prompts related to cross-view discrepancy resolution or representative plane selection during instruction.

### 2.4 Course design and case development

#### 2.4.1 Course workflow and instructional standardization

Each session followed three sequential phases: (1) a 20-min review of TI-RADS scoring principles and key feature definitions; (2) a 50-min case-analysis phase using a common small-group discussion format with faculty-guided discussion of image interpretation and final categorization; and (3) a 20-min faculty synthesis of key decision rules, common interpretive errors, and practical principles for assigning a final TI-RADS category. During these phases, each group discussed 20 teaching cases, and the same case set was used in both study arms.

To minimize contamination while preserving instructor consistency, the control session was delivered before the intervention session, and the instructor was not informed of the intervention-specific prompting strategy until completion of the control session.

#### 2.4.2 AI system and input standardization

AI analysis was performed using the AI-SONIC™ ultrasound intelligent auxiliary diagnostic system (model AI-SONIC-DE; version V5.5.8.2; Demetics Medical Technology Ltd., Hangzhou, China) with default settings. AI-generated feature recognition and TI-RADS categorization were used as produced by the system. Expert review was limited to confirming that each output matched the submitted image, without revising the AI result to align with expert judgment.

#### 2.4.3 Multi-view AI output generation and instructional use

For each teaching case, each available image was submitted separately to the AI system as a plane-level input. Multi-view AI output variability was operationally defined as any within-case difference in AI-generated feature assessment or TI-RADS category across submitted views.

During the intervention session, images and their corresponding plane-level AI outputs were reviewed together on a case-by-case basis. AI output was used as a discrepancy signal rather than as an answer key. The instructor applied the prespecified prompt sequence to guide learners through four steps: comparison of transverse and longitudinal views; identification of plausible sources of output variability, including non-representative views, boundary ambiguity, and imaging artifact; selection of the most representative plane for final categorization; and justification of a final TI-RADS category based on evidence integrated across views.

Throughout this process, the final TI-RADS decision remained the learner's judgment rather than an automatic adoption of a single AI output. Figure 1 presents the intervention as a transition from AI-derived plane-level variability to faculty-guided comparison and, ultimately, independent learner judgment. Accordingly, AI output was used as a discrepancy signal rather than as an answer key.

#### 2.4.4 Case development and quality control

Training and assessment cases were sampled from independent case pools, with equal proportions of cases with and without multi-view AI output variability in each. For outcome assessment, each participant completed 20 non-overlapping thyroid nodule cases at each time point (T0, T1, and T2), yielding 60 assessment cases per participant across the study. The three assessment case sets were matched *a priori* with respect to TI-RADS category distribution and the proportions of key suspicious ultrasound features, including irregular margins, nonparallel shape, and punctate echogenic foci.

Each case consisted of 3–4 static images acquired during the same examination session from the same nodule and included at least one transverse and one longitudinal view. The representative imaging plane for each case was predefined by expert consensus. During case development, the expert panel identified at least one image as the representative plane for each case. The representative plane had to meet at least two of the following: clearly visualized margins with minimal artifact, visualization of the maximal or near-maximal lesion diameter, stable demonstration of diagnostically relevant ultrasound features, and suitability for standardized measurement.

Expert reference standards for final TI-RADS categorization were established by two senior thyroid ultrasound specialists. Each specialist independently reviewed every assessment case. Any disagreements were resolved through structured discussion to reach unanimous consensus.

### 2.5 Assessment strategy and outcomes

Assessments were conducted at baseline (T0), immediately after the instructional session (T1), and at 4-week follow-up (T2). The outcome structure was prespecified to distinguish longitudinal performance outcomes from proximal post-instruction reasoning indicators.

At the prespecified time points, participants completed case-based TI-RADS interpretation tasks and self-efficacy assessment according to the schedule described below. For the primary accuracy outcome at T1, only the initial TI-RADS category assigned before exposure to AI outputs was used. AI outputs were displayed afterward only to generate discordant cases for the exploratory justification task.

#### 2.5.1 Primary outcome

The primary outcome was participant-level TI-RADS interpretation accuracy at T0, T1, and T2, defined as the number of correctly categorized cases out of 20 relative to expert consensus at each time point.

#### 2.5.2 Secondary outcomes

***Inter-learner consistency:*** Agreement in TI-RADS categorization among learners within each study group, evaluated at T0, T1, and T2.

***Representative plane hit score (T1 only):*** The number of cases in which a participant correctly selected the expert-defined representative plane out of 20 assessment cases. This outcome was prespecified as a proximal post-instruction indicator of whether the intervention improved view selection for final TI-RADS categorization.

***Self-efficacy:*** A 7-item Likert-scale measure administered at T0 and T1, analyzed as the participant-level mean item score (range, 1–7).

#### 2.5.3 Exploratory outcomes

***Justification quality (T1 only):*** At T1, participants first assigned a TI-RADS category to each case on the basis of the ultrasound images alone, without access to AI results. They were then shown the corresponding AI output. Cases in which their rating was inconsistent with the AI-derived categorization were classified as discordant. For these discordant cases, participants were required to provide a brief justification for the discrepancy. Justification quality was scored on a 0–3 rubric reflecting the extent to which the participant linked the final categorization to specific image features. In addition, justification quality for all eligible discordant cases was independently scored by two blinded raters using prespecified scoring rules. Disagreements were resolved by discussion until consensus was reached.

### 2.6 Statistical analysis

All analyses were conducted at the participant level unless otherwise specified. Categorical variables were presented as counts and percentages. Continuous variables were summarized as mean ± standard deviation (SD) or median (range), as appropriate, according to their distribution.

Accuracy and self-efficacy were analyzed using a linear mixed-effects model; between-group differences at each time point were evaluated using *post hoc* model-based contrasts, whereas the difference in change over time between groups was evaluated by the group-by-time interaction. Time was modeled as a categorical variable. Between-group differences in Representative plane hit score were compared using the independent-samples *t* test or the Wilcoxon rank-sum test, as appropriate. Justification quality was treated as an exploratory analysis because it was assessed only at T1 and only in eligible discordant cases, rather than uniformly across all participants and time points. Between-group differences in justification quality were evaluated using a mixed-effects ordinal logistic regression model with study group as a fixed effect and participant as a random intercept. Inter-rater reliability for justification quality scoring was assessed using the intraclass correlation coefficient (ICC). An ICC of at least 0.75 was considered acceptable. Inter-learner consistency in TI-RADS categorization was analyzed as a group-level outcome using Gwet's AC2 with ordinal weights. Between-group differences at each time point and within-group changes across time points were estimated by bootstrap resampling with 2,000 iterations. Statistical significance was inferred when the 95% confidence interval (CI) did not include zero.

Statistical analyses were performed using R (version 4.3.2). All hypothesis tests were two-sided with a significance threshold of *P* < 0.05; for bootstrap-estimated AC2 differences, statistical significance was inferred when the 95% CI did not include zero.

## 3 Results

### 3.1 Participant flow and baseline characteristics

#### 3.1.1 Enrollment and allocation

**Figure 2:**
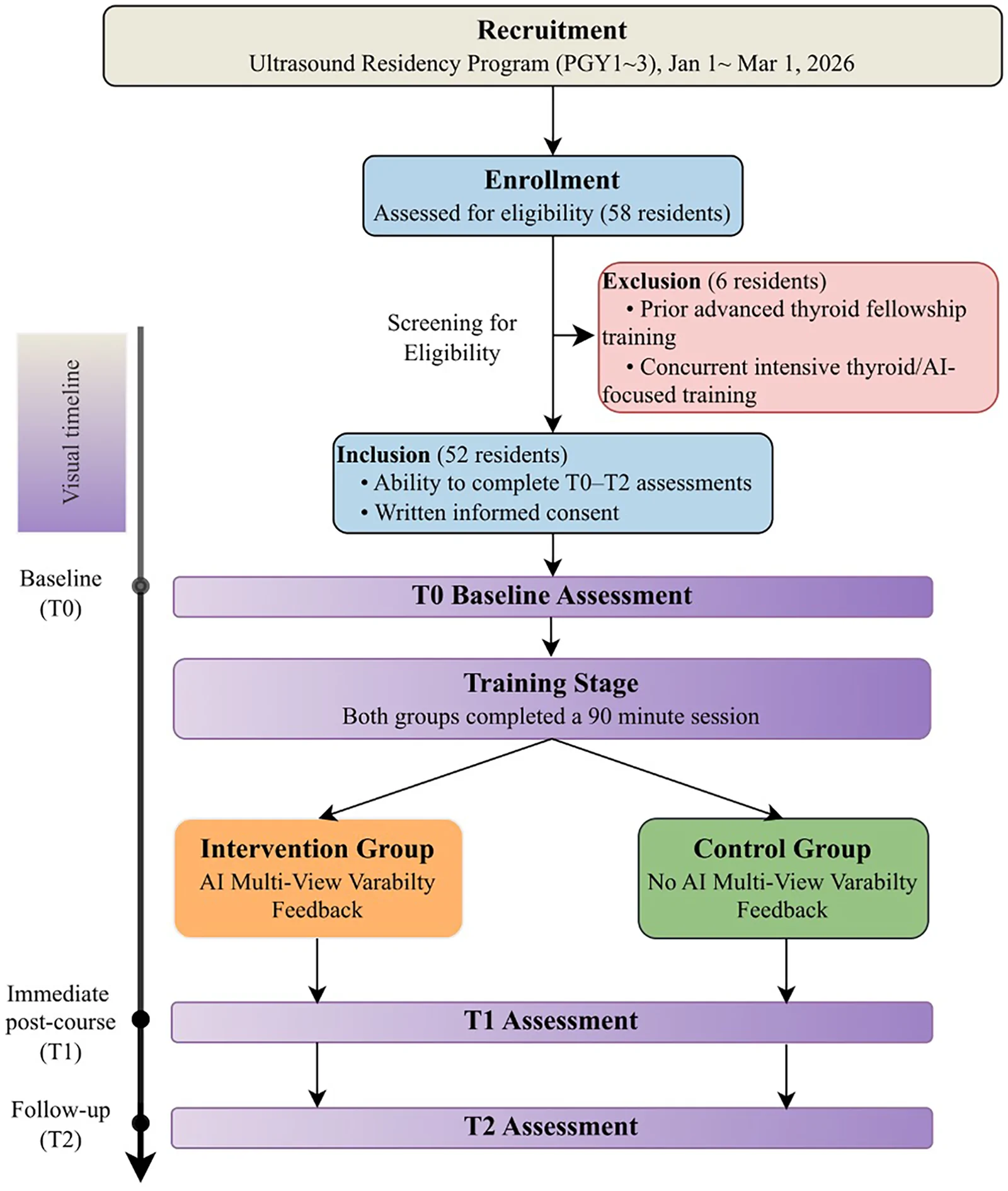
Study flowchart and assessment timeline. Of 58 residents assessed for eligibility, 6 were excluded and 52 were randomized 1:1 to the intervention (*n* = 26) and control (*n* = 26) groups. All participants completed a 90-min teaching session and assessments at baseline (T0), immediately after teaching (T1), and 4-week follow-up (T2), with no loss to follow-up. The intervention group received AI multi-view output-variability feedback; the control group did not. PGY, postgraduate year; T0, baseline; T1, immediately after teaching; T2, 4-week follow-up.

A total of 52 residents were randomized, with 26 assigned to each group. All randomized participants received the allocated teaching session and were included in the primary analysis. No participants were lost to follow-up at T1 or T2. The participant flow diagram is shown in Figure 2.

#### 3.1.2 Baseline comparability

Baseline characteristics were comparable between groups. No significant between-group differences were found in age, sex, and PGY distribution (all *P* > 0.05; Table 1).

**Table 1:** Baseline characteristics of participants by study group.

Characteristic	All	Intervention	Control	*P*-value
Resident, n	52	26	26	
Age	25.50 (25.00, 26.00)	25.00 (25.00, 26.00)	25.00 (25.00, 26.00)	0.923
Sex, *n* (%)				1.000
Male	28 (53.8%)	14 (53.8%)	14 (53.8%)	
Female	24 (46.2%)	12 (46.2%)	12 (46.2%)	
PGY (1–3), *n* (%)				0.782
1–2	26 (50.0%)	14 (53.8%)	12 (46.2%)	
3	26 (50.0%)	12 (46.2%)	14 (53.8%)	

Values are presented as *n* (%) or median (range), as appropriate. PGY, postgraduate year; in this study, PGY is used to indicate residency year (e.g., PGY-1, first-year resident).

### 3.2 Primary outcome: TI-RADS interpretation accuracy

The group-by-time interaction was significant [*F* (2, 100.0) = 10.86, *P* < 0.001], indicating different accuracy trajectories between groups. At T0, accuracy did not differ between groups (*β* = 0.08, 95% CI: −0.74 to 0.89, *P* = 0.851). At T1, the intervention group showed higher accuracy than the control group (*β* = 1.69, 95% CI: 0.88–2.51, *P* < 0.001). At T2, accuracy remained numerically higher in the intervention group, but the between-group difference was not significant (*β* = 0.73, 95% CI: −0.08 to 1.55, *P* = 0.078).

**Figure 3:**
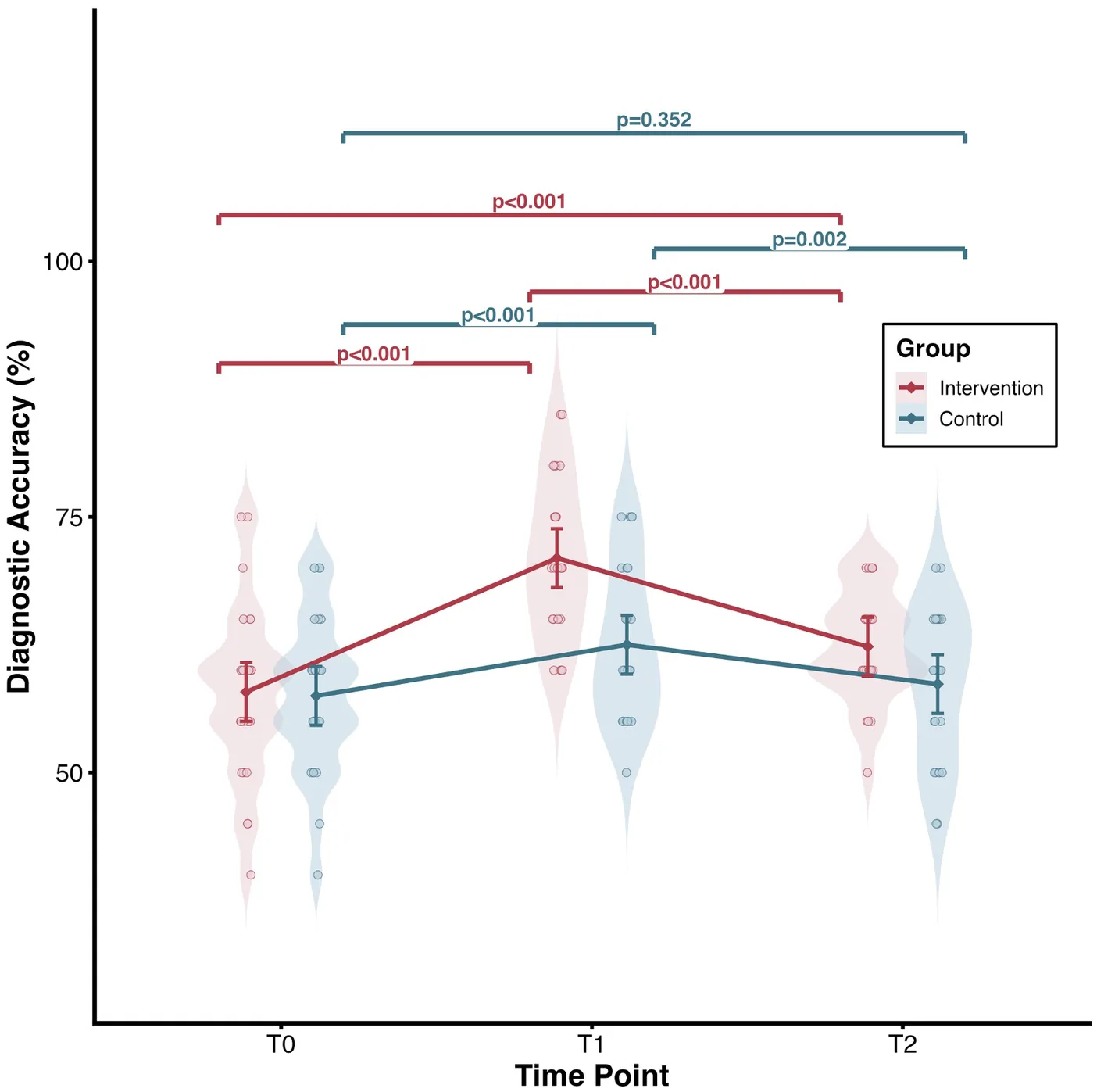
Participant-level TI-RADS interpretation accuracy across T0, T1, and T2. Violin plots show the distribution of participant-level diagnostic accuracy at baseline (T0), immediately after teaching (T1), and 4-week follow-up (T2) in the intervention (red) and control (blue) groups. For the intervention group, all three contrasts were significant (T0 vs. T1, T1 vs. T2, and T0 vs. T2; all *P* < 0.001). For the control group, accuracy increased from T0 to T1 (*P* < 0.001), decreased from T1 to T2 (*P* = 0.002), and did not differ between T0 and T2 (*P* = 0.352).

Within-group analyses showed significant improvement from T0 to T1 in both the intervention group (*β* = 2.62, 95% CI: 2.13–3.11, *P* < 0.001) and the control group (*β* = 1.00, 95% CI: 0.511–1.49, *P* < 0.001). From T1 to T2, accuracy declined in both groups. At T2, the intervention group remained above baseline (*β* = 0.89, 95% CI: 0.40–1.37, *P* < 0.001), whereas the control group did not differ from baseline (*β* = 0.23, 95% CI: −0.26 to 0.72, *P* = 0.352). Figure 3 shows the distribution of participant-level accuracy scores at each time point in both groups.

### 3.3 Secondary outcomes and exploratory analysis

The results for the primary and secondary outcomes are summarized in Table 2.

**Table 2:** Primary and key secondary outcomes across time points (T0–T2) in the intervention and control groups.

Outcome	Time	Intervention	Control	Difference (95% CI)	*P*
Accuracy	T0	11.58	11.50	0.08 (−0.74, 0.89)	0.851
	T1	14.19	12.50	1.69 (0.88, 2.51)	<0.001
	T2	12.46	11.73	0.73 (−0.08, 1.55)	0.078
Inter-learner consistency	T0	0.71	0.70	0.01 (−0.01, 0.02)	0.161
	T1	0.80	0.73	0.08 (0.04, 0.11)	<0.001
	T2	0.78	0.69	0.09 (0.06, 0.12)	<0.001
Representative plane hit score	T1	15.00 (12.00, 17.00)	13.00 (11.00, 15.00)	2.00 (1.00, 3.00)	<0.001
Self-efficacy	T0	3.45 (2.70, 4.00)	3.50 (2.60, 3.95)	−0.05 (−1.25, 1.40)	0.941
	T1	4.85 (4.15, 5.40)	4.70 (4.00, 5.00)	0.25 (−0.85, 1.40)	<0.001

CI, confidence interval; PGY, postgraduate year; T0, baseline; T1, immediately after the instructional session; T2, 4-week follow-up.

#### 3.3.1 Representative plane hit score

At T1, the intervention group had a higher Representative plane hit score than the control group. Because the control group showed non-normality on the Shapiro–Wilk test (*P* = 0.022), between-group comparison used the Wilcoxon rank-sum test. Median hit score was 15.00 (12.00, 17.00) in the intervention group and 13.00 (11.00, 15.00) in the control group, with a significant between-group difference (*P* < 0.001).

#### 3.3.2 Inter-learner consistency

Inter-learner consistency in TI-RADS categorization, measured by Gwet's second-order agreement coefficient (AC2) with ordinal weights, was 0.71 in the intervention group and 0.70 in the control group at T0, 0.80 and 0.73 at T1, and 0.78 and 0.69 at T2, respectively. Between-group bootstrap comparisons showed no significant difference at T0 (ΔAC2 = 0.01, 95% CI: −0.01 to 0.02, *P* = 0.161), but significantly higher consistency in the intervention group at T1 (ΔAC2 = 0.08, 95% CI: 0.04–0.11, *P* < 0.001) and T2 (ΔAC2 = 0.09, 95% CI: 0.06–0.12, *P* < 0.001).

**Figure 4:**
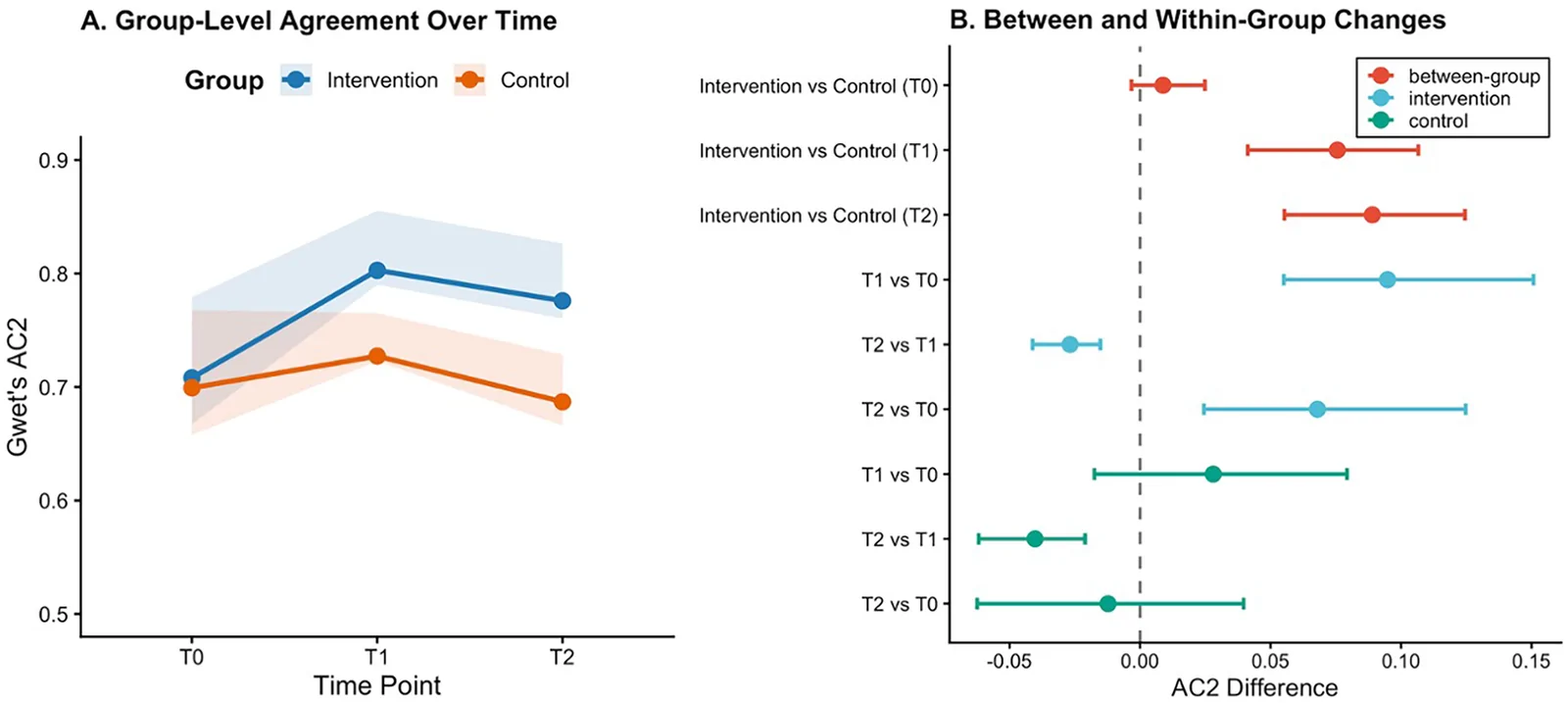
Inter-learner consistency (Gwet's AC2) across T0, T1, and T2. **(A)** AC2 estimates with 95% confidence intervals are shown across T0–T2 for the intervention (blue) and control (orange) groups. **(B)** Points and horizontal bars show bootstrap-estimated between-group (red), within-intervention (light blue), and within-control (teal) differences with 95% confidence intervals from 2,000 resamples. Statistical significance was inferred when the 95% confidence interval (CI) did not include zero.

Within-group analyses showed that AC2 increased from T0 to T1 in the intervention group (ΔAC2 = 0.09, 95% CI: 0.06–0.15, *P* < 0.001), declined from T1 to T2 (ΔAC2 = −0.03, 95% CI: −0.04 to −0.02, *P* < 0.001), but remained higher at T2 than at T0 (ΔAC2 = 0.07, 95% CI: 0.02–0.12, *P* < 0.001). In the control group, AC2 did not change from T0 to T1 (ΔAC2 = 0.03, 95% CI: −0.02 to 0.08, *P* = 0.227), decreased from T1 to T2 (ΔAC2 = −0.04, 95% CI: −0.06 to −0.02, *P* = 0.009), and did not differ between T2 and T0 (ΔAC2 = −0.01, 95% CI: −0.06 to 0.04, *P* = 0.689). The temporal patterns of inter-learner consistency in the two groups are shown in Figure 4.

#### 3.3.3 Self-efficacy

At T0, self-efficacy did not differ between groups (*P* = 0.941). The group-by-time interaction was significant (*β* = 0.27, SE = 0.09, 95% CI: 0.09–0.45, *P* = 0.006), indicating a greater increase in the intervention group. *Post hoc* comparisons showed no between-group difference at T0 (*P* = 0.941), but higher self-efficacy in the intervention group at T1 (*P* < 0.001). Within-group increases from T0 to T1 were significant in both groups (both *P* < 0.001).

#### 3.3.4 Exploratory analysis: justification quality

At T1, 239 discordant participant-case responses were identified, including 165 in the intervention group and 74 in the control group. Inter-rater reliability was acceptable (ICC: 0.88, 95% CI: 0.84–0.90). Mixed-effects ordinal logistic regression showed a more favorable justification quality distribution in the intervention group than in the control group (OR: 10.26, 95% CI: 5.36–19.67; *P* < 0.001).

## 4 Discussion

This randomized controlled trial found that, compared with time-matched conventional teaching, a structured micro-course using multi-view AI output variability as a teaching resource improved immediate TI-RADS interpretation accuracy and was also associated with a higher representative plane hit score, greater inter-learner consistency, and larger gains in self-efficacy. At 4 weeks, inter-learner consistency remained higher in the intervention group, but the between-group difference in accuracy was no longer statistically significant. The findings therefore support an immediate educational benefit, whereas a sustained advantage in interpretation accuracy was not established after a single session.

One possible explanation is that the intervention helped externalize interpretive processes that are often difficult to teach directly. TI-RADS classification across image planes depends not only on applying scoring rules, but also on comparing views, identifying the most representative plane, and integrating partially discordant visual information (4, 5). Such processes are partly perceptual and are not always fully formalized in verbal rules. When these otherwise tacit steps are made more explicit during instruction, learners may be better able to understand how expert judgments are formed and to apply similar reasoning in subsequent cases (6). This interpretation is also consistent with the higher Representative plane hit score observed in the intervention group.

The findings can also be understood from a clinical reasoning perspective. In many educational settings, reasoning is only indirectly addressed, with greater attention given to the final answer than to the process used to reach it (18). In contrast, the present intervention directed attention to comparison, prioritization, and evidence integration across views. This approach aligns with the view that clinical reasoning should be taught explicitly rather than left implicit (9). It also reflects a cognitive apprenticeship orientation, in which expert interpretive processes are made more explicit, discussable, and learnable through guided instruction (10, 19, 20).

The greater increase in self-efficacy in the intervention group is also noteworthy. In medical education, self-efficacy is not only a learner perception but a meaningful indicator of confidence in performing task-specific judgments (21, 22). In this study, the intervention may have strengthened self-efficacy by making the reasoning process more transparent and manageable. Rather than relying only on the final category, learners were given a clearer basis for how to compare planes, select the most informative view, and justify a TI-RADS decision. This may have increased their sense of control over the interpretive task.

More broadly, these findings suggest that AI in ultrasound education may have value beyond reader assistance. The educational use of AI has also received growing attention (16, 23, 24). Most prior work has focused on whether AI improves diagnostic performance or supports less experienced readers in clinical interpretation. In contrast, the present study used multi-view AI output variability as a teaching resource. Its educational value may lie less in providing a single correct-looking output than in making interpretive differences visible enough to support guided reasoning. This perspective may be especially relevant in settings where expert teaching resources are limited, because it offers a way to turn AI-generated variability into material for structured instruction.

However, these mechanistic interpretations should remain tentative. The intervention combined multi-view AI output variability with structured prompting, faculty-guided reasoning, representative-plane selection, and explicit justification of decisions. Because these elements were delivered as an integrated educational package, the observed effects cannot be attributed exclusively to AI output variability or separated from the contribution of established instructional strategies. The proposed role of AI-generated variability in externalizing cross-plane reasoning should be regarded as a plausible explanatory hypothesis.

The temporal pattern may indicate that the intervention accelerated initial learning without fully consolidating the new reasoning strategy after one 90-min session. Accuracy declined from T1 to T2 in both groups, whereas the intervention group retained higher inter-learner consistency at T2. These different trajectories suggest that learners may have retained a more consistent approach to categorization even though the between-group accuracy advantage diminished. Spaced practice, booster case discussions, or repeated exposure to cross-view discrepancies may help reinforce independent accuracy, but this possibility requires prospective evaluation.

Several limitations should be considered. First, this was a single-center study with a modest sample size, which may limit generalizability. Second, because the educational content differed visibly between groups, blinding of participants and the instructor was not feasible, and expectancy or performance effects cannot be excluded. To minimize between-instructor variability, both sessions were delivered by the same instructor using standardized materials and a prespecified facilitation checklist. Nevertheless, some of the observed between-group differences may still have reflected instructor-related effects. The study also did not measure cognitive load, obtain qualitative feedback from learners, or directly examine changes in clinical reasoning processes. Therefore, the mechanisms underlying the observed effects remain uncertain. Third, follow-up was limited to 4 weeks, and no later assessments were performed. Thus, the study cannot determine whether the immediate accuracy gain would persist, further decay, or be restored by spaced or booster instruction, and longer follow-up with repeated assessments is needed. In addition, justification quality was analyzed only in eligible discordant cases at T1 and should be interpreted as exploratory. Finally, the study did not directly examine whether learners transferred these gains to real-time scanning or broader clinical performance.

## 5 Conclusions

In this randomized controlled trial, a structured micro-course using multi-view AI output variability as a teaching resource improved immediate TI-RADS interpretation accuracy, representative plane hit score, inter-learner consistency, and self-efficacy among ultrasound residents. Although inter-learner consistency remained higher at 4 weeks, the study did not establish a sustained between-group advantage in interpretation accuracy. Because the intervention integrated AI output variability with structured prompting and faculty-guided reasoning, these effects should be attributed to the micro-course as a whole rather than to AI output variability as an independent causal mechanism. Overall, the findings support the short-term educational value of this integrated approach, while repeated or booster sessions warrant investigation as potential strategies for improving longer-term retention.

## Data Availability

The data analyzed in this study is subject to the following licenses/restrictions: The de-identified data supporting the conclusions of this article are available from the first author upon reasonable request, subject to institutional and ethical approval. Requests to access these datasets should be directed to Fu Chao, fuchao3380@163.com.
