# Supplementary material for: Multi-view AI output variability as a teaching resource in a micro-course for thyroid TI-RADS interpretation among ultrasound residents: a randomized controlled trial
Source: Front Med (Lausanne). 2026 Aug 26;13:1868341. doi: 10.3389/fmed.2026.1868341 (PMC13553353; doi:10.3389/fmed.2026.1868341)
Supplement: Supplementary file 1 [file Table1.docx]

7-point Likert scale options and anchors

| **Response value** | **Anchor** | **Interpretive description** |
| --- | --- | --- |
| 1 | Not confident at all | Basically unable to complete the task |
| 2 | Slightly confident | Unable to complete the task in most situations |
| 3 | Somewhat unconfident | Able to complete the task only with substantial help |
| 4 | Neutral | Sometimes able to complete the task, sometimes not |
| 5 | Fairly confident | Able to complete the task in most situations |
| 6 | Very confident | Able to complete the task in almost all situations |
| 7 | Completely confident | Able to complete the task consistently and independently |
