# Supplementary material for: Multi-view AI output variability as a teaching resource in a micro-course for thyroid TI-RADS interpretation among ultrasound residents: a randomized controlled trial
Source: Front Med (Lausanne). 2026 Aug 26;13:1868341. doi: 10.3389/fmed.2026.1868341 (PMC13553353; doi:10.3389/fmed.2026.1868341)
Supplement: Supplementary file 2 [file Table2.docx]

Scoring rubric

**0 points:** No explanation, or purely subjective judgment.
Example: “I think it is 4B.” / “The AI is inaccurate.”

**1 point:** The discrepancy is identified, but no mechanism or evidence is provided.
Example: “The AI interpreted the margin as unclear, but I considered it clear.”

**2 points:** The discrepancy is identified, and a reasonable explanation or mechanism is provided.
Example: “The AI misclassified the finding as microcalcification because acoustic shadowing at an oblique angle affected the interpretation.”

**3 points:** The discrepancy is identified, the explanation or mechanism is provided, and the relevant imaging plane used as evidence is clearly specified.
Example: “The selected plane was not representative, which made the margin appear indistinct; on the longitudinal plane, the margin appeared irregular.”
